# COVID-19 vaccine effectiveness against severe disease from SARS-CoV-2 Omicron BA.1 and BA.2 subvariants – surveillance results from southern Sweden, December 2021 to March 2022

**DOI:** 10.2807/1560-7917.ES.2022.27.18.2200322

**Published:** 2022-05-05

**Authors:** Jonas Björk, Carl Bonander, Mahnaz Moghaddassi, Magnus Rasmussen, Ulf Malmqvist, Malin Inghammar, Fredrik Kahn

**Affiliations:** 1 Clinical Studies Sweden, Forum South, Skåne University Hospital, Lund, Sweden; 2 Division of Occupational and Environmental Medicine, Lund University, Lund, Sweden; 3 School of Public Health and Community Medicine, Institute of Medicine, University of Gothenburg, Gothenburg, Sweden; 4 Social Medicine and Global Health, Department of Clinical Sciences Malmö, Lund University, Malmö, Sweden; 5 Department of Clinical Sciences Lund, Section for Infection Medicine, Skåne University Hospital, Lund University, Lund, Sweden

**Keywords:** SARS-CoV-2 vaccine effectiveness, epidemiological surveillance, variant of concern

## Abstract

We compared vaccine effectiveness against severe COVID-19 between December 2021 and March 2022 when Omicron BA.1 and BA.2 were the dominating SARS-CoV-2 variants in Scania county, Sweden. Effectiveness remained above 80% after the transition from BA.1 to BA.2 among people with at least three vaccine doses but the point estimate decreased markedly to 54% among those with only two doses. Protection from prior infection was also lower after the transition to BA.2. Booster vaccination seems necessary to maintain sufficient protection.

The severe acute respiratory syndrome coronavirus 2 (SARS-CoV-2) variant of concern (VOC) Omicron (Phylogenetic Assignment of Named Global Outbreak (Pango) lineage designation B.1.1.529) has two genetically divergent subvariants, BA.1 and BA.2 that emerged in late 2021 and early 2022, respectively. A comparison of secondary attack rates from Denmark suggests that the BA.2 subvariant carries a transmission advantage compared with BA.1 [1], which may explain why BA.2 has rapidly replaced BA.1 as the dominant subvariant in several countries. A study from the United Kingdom suggests comparable vaccine protection against symptomatic disease from BA.2 as for BA.1 [2]. However, few studies have compared the protection against severe disease from these two Omicron subvariants. Recent findings from Qatar suggested lower vaccine protection against hospitalisations and deaths from BA.2 but with a very wide confidence interval [3].

We aimed to compare COVID-19 vaccine effectiveness (VE) against severe disease from the Omicron BA.1 and BA.2 subvariants, in Scania county, southern Sweden, a region with routine sequencing of samples of infected cases.

## Studied population and period

The study cohort included all individuals residing in Scania county (Skåne), southern Sweden, on 27 December 2020 (baseline) when COVID-19 vaccinations began (n = 1,384,531) [4,5]; this cohort was followed longitudinally for positive SARS-CoV-2 tests, hospitalisations and assessment of disease severity. Individuals who died or moved away from the region were censored on the date of death or relocation. 

We used available data from routine sequencing of samples of infected cases in Scania county (Supplementary Table S1) to define three specific follow-up periods for the evaluation of VE in the present study, during which the proportion of BA.2 surpassed BA.1 and the Omicron the Delta (Pango lineage designation B.1.617.2) VOC. These calendar periods were: (i) Omicron BA.1 as the dominating VOC, week 52 2021 – week 1 2022 (respective proportions of SARS VOCs – BA.1: 60%, Delta: 25%, BA.2: 15%), (ii) transition period, week 2–3 2022 (BA.1: 47%, Delta: 4.5%, BA.2: 49%), and (iii) Omicron BA.2 as the dominating VOC, week 4–11 2022 (until date of data extraction on 15 March – BA.1: 17%, Delta: 0.5%, BA.2: 82%).

## Data extraction and case definition

Demographic and clinical data collected from national and regional register holders were linked using the personal identification number assigned to all Swedish residents. Weekly updates on vaccination date, type of vaccine and dose were obtained from the National Vaccination Register, and data on COVID-19 cases (defined by a positive SARS-CoV-2 test result) from the electronic system SMINet, both kept at the Public Health Agency of Sweden. Registration in SMINet included positive PCR tests, but also positive rapid antigen tests used in healthcare and at private testing facilities. Regional health registers were used as complementary data sources to rapidly provide data on positive tests, and to assess comorbidities and disease outcomes.

Comorbidities were defined from diagnoses in inpatient or specialised care at any time point during the 5 years before baseline in the following disease groups: cardiovascular diseases, diabetes or obesity, kidney or liver diseases, respiratory diseases, neurological diseases, cancer or immunosuppressed states, and other conditions and diseases (Down syndrome, HIV, sickle cell anaemia, drug addiction, thalassaemia or mental health disorder) (for a detailed list see Supplementary Table S2: Classification of comorbidities). The number of comorbidities in these groupings was counted and used in the analyses. 

A severe COVID-19 case was defined as a case who was hospitalised for at least 24 h from 5 days before until 14 days after a positive SARS-CoV-2 test and required oxygen supply (≥ 5 L/min) or admittance to an intensive care unit (ICU).

## Vaccine effectiveness

We used continuous density case–control sampling [6] nested within the study cohort together with conditional logistic regression to estimate VE against severe COVID-19. For each severe case, 10 controls without a positive SARS-CoV-2 test the same week as the case or 90 days prior were randomly selected from the underlying study cohort, matched with respect to sex and age (5-year groups). Only vaccine doses obtained more than 7 days before the positive test of the case were counted in the analyses. Comirnaty (BNT162b2 mRNA, BioNTech-Pfizer) was most frequently used vaccine type, representing 77% of all administrated doses in the study cohort; Spikevax (mRNA-1273, Moderna) and Vaxzevria (ChAdOx1 nCoV-19, Oxford-AstraZeneca) were also used in the vaccination program.

**Figure 1:**
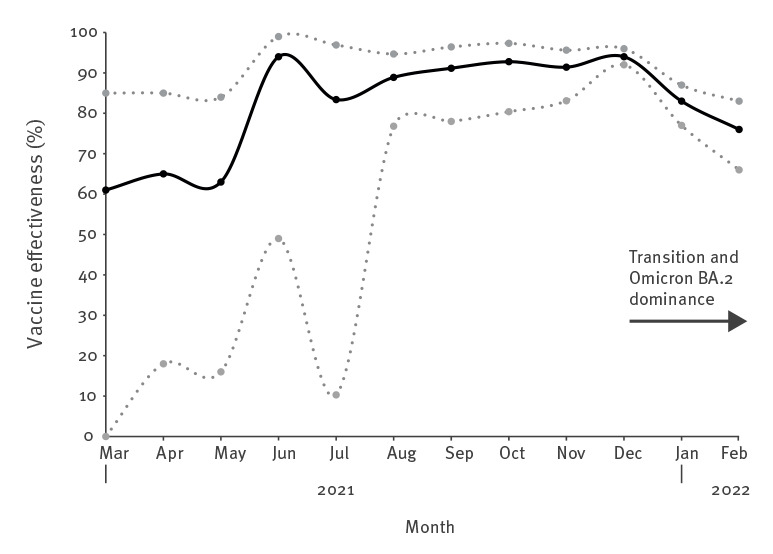
Surveillance of vaccine effectiveness after at least two doses against severe COVID-19, in Scania, Sweden, March 2021–February 2022 (n = 1,381 cases; n = 13,810 controls)

A total of 593 severe COVID-cases occurred during the study period, corresponding to 65, 78 and 56 cases weekly during the Omicron BA.1, the transition and Omicron BA.2 periods, respectively (Table 1). Severe cases during the BA.2 period were older and had a more even sex distribution versus the BA.1 period. The overrepresentation of individuals born abroad among severe cases observed during BA.1 was less marked during BA.2, but the presence of comorbidities was similar. The monthly surveillance shows that population protection against severe COVID-19 after at least two doses was high before the follow-up periods of the present study started (median VE: 89% during March–November 2021) (Figure 1). Population protection remained stable also during the Delta and Omicron BA.1 dominance in December 2021, whereas the transition from Omicron BA.1 to BA.2 in January–February 2022 was associated with a decline in protection.

**Table 1:** Characteristics of severe COVID-19 cases (n = 593) and controls (n = 5,930), stratified by follow-up period for monitoring of vaccine effectiveness during SARS-CoV-2 Omicron variant dominance, Scania, Sweden, 2021 week 52–2022 week 11

Characteristics	Follow-up period											
	Omicron BA.1 2021 w52–2022 w1				Transition 2022 w2–3				Omicron BA.2 2022 w4–11			
	Cases (n = 129)		Controls (n = 1,290)		Cases (n = 156)		Controls (n = 1,560)		Cases (n = 308)		Controls (n = 3,080)	
Weekly case rate, mean (n)	65		NA		78		NA		56^a^		NA	
Age group (years)	%	n	%	n	%	N	%	n	%	n	%	n
0–17	3.1	4	3.6	47	5.1	8	4.3	67	5.2	16	5.1	156
18–39	16	21	15	198	9.0	14	10	157	12	37	12	364
40–64	30	39	30	392	23	36	24	367	15	47	17	523
≥ 65	50	65	51	653	63	98	62	969	68	208	66	2,037
Sex												
Female	37	48	37	480	38	60	38	600	50	153	50	1,530
Male	63	81	63	810	62	96	62	960	50	155	50	1,550
Born abroad	46	60	22	277	32	50	20	309	23	70	18	561
Civil status												
Married	45	58	47	611	36	56	48	746	37	113	47	1,460
Widow/widower	8.5	11	8.8	114	15	24	11	176	16	48	15	462
Divorced	21	27	15	197	21	33	17	259	23	70	14	443
Single	26	33	28	368	28	43	24	379	25	77	23	715
Comorbidities												
0	36	46	63	816	38	59	58	905	33	103	56	1,736
1	26	33	21	273	26	40	23	362	25	78	25	758
≥ 2	39	50	16	201	36	57	19	293	41	127	19	586
Vaccine doses												
0	54	70	14	185	42	65	13	201	27	82	13	394
1	3.1	4	1.8	23	4.5	7	1.8	28	4.5	14	1.6	50
2	18	23	37	481	28	44	29	459	20	62	17	519
3	25	32	47	601	26	40	56	872	49	150	68	2,108
4	0.0	0	0.0	0	0.0	0	0.0	0	0.0	0	0.3	9
Vaccine type^b^												
Comirnaty	74	41	72	775	71	60	70	934	73	155	69	1,825
Spikevax	3.6	2	6.7	73	13	11	4.9	65	4.7	10	4.3	114
Vaxzevria	3.6	2	2.3	25	4.8	4	2.0	27	2.4	5	1.0	26
Mixed	18	10	19	209	11	9	23	305	20	42	26	671
Time since last vaccine dose^b^ (months)												
0–3	54	30	60	650	43	36	66	884	38	80	56	1,470
3–6 months	20	11	27	288	27	23	19	256	43	92	35	933
≥ 6 months	26	14	13	144	30	25	14	191	19	40	8.8	233
Prior SARS-CoV-2 infection	2.3	3	8.8	114	3.8	6	8.2	128	5.8	18	8.1	250
At least two doses or prior SARS-CoV-2 infection	43	56	85	1,101	54	85	87	1,353	70	216	87	2,675

COVID-19: coronavirus disease; SARS-CoV-2: severe acute respiratory syndrome coronavirus 2.

^a^ Last two weeks of the Omicron BA.2 was not included in the calculation of weekly case rate in order to limit the impact of delay in manifestation of severe cases.

^b^ Only persons with at least two doses.

A severe COVID-19 case was defined as a case who was hospitalised for at least 24 h from 5 days before until 14 days after a positive SARS-CoV-2 test and required oxygen supply (≥ 5 L/min) or admittance to an intensive care unit (ICU). For each severe case, 10 controls without a positive test the same week as the case or 90 days prior were randomly selected from the underlying study cohort, matched with respect to sex and age (5-year groups).

COVID-19: coronavirus disease.

The following vaccines were used during the study period: Comirnaty (BNT162b2 mRNA, BioNTech-Pfizer), Spikevax (mRNA-1273, Moderna), Vaxzevria (ChAdOx1-S, AstraZeneca). Estimated vaccine effectiveness obtained from conditional logistic regression for age and sex matched case and controls (1:10). Solid black curves indicate vaccine effectiveness. Grey dotted lines represent 95% confidence intervals.

**Figure 2:**
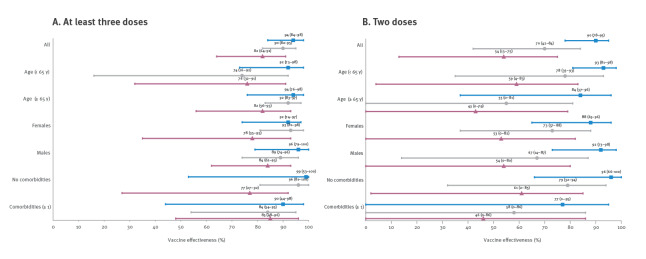
Vaccine effectiveness against severe COVID-19 in each follow-up period during SARS-CoV-2 Omicron variant dominance, Scania, Sweden, 2021 week 52 – 2022 week 11 (n = 593 cases; n = 5,930 controls)

COVID-19: coronavirus disease; SARS-CoV-2: severe acute respiratory syndrome coronavirus 2.

In more detailed analyses of the change in VE, numbers of vaccine doses were grouped as 0–1, 2 or at least 3 and we adjusted for the number of comorbidities and infection at least 90 days before the case date. Whereas the VE after at least three doses remained above 80% throughout the study period, VE after two doses declined substantially from 90% (95% confidence interval (CI)): 78–95) during Omicron BA.1 dominance to 54% (95% CI: 13–75) during BA.2 dominance (Figure 2 and Supplementary Table S3: Vaccine effectiveness after at least three or two doses against severe COVID-19). This decline was consistently noted across subgroups of age, sex and comorbidities.

Omicron BA.1 period 2021 w52–2022 w1 (blue boxes; n=129 cases; n=1,290 controls), transition period 2022 w2–3 (gray circles; n = 156 cases; n = 1,560 controls), Omicron BA.2 period 2022 w4–11 (pink triangles; n = 308 cases; n = 3,080 controls) after at least three (A) or two doses (B) of COVID-19 vaccine. Shapes represent point estimates and lines 95% confidence intervals.

The following vaccines were used during the study period: Comirnaty (BNT162b2 mRNA, BioNTech-Pfizer), Spikevax (mRNA-1273, Moderna), Vaxzevria (ChAdOx1-S, AstraZeneca).

Estimates were obtained from conditional logistic regression for age and sex matched case and controls (1:10), and with adjustment for comorbidities (0, 1, ≥ 2) and infection at least 90 days prior the case date. Results are presented overall and stratified by age, sex and comorbidities.

We also compared the protection from vaccination and infection history (Table 2). Surviving a prior SARS-CoV-2 infection offered similar protection against new onset of severe COVID-19 as two or three vaccine doses both during Omicron BA.1 dominance and the transition period. However, the protection associated with prior infection and at most two doses decreased markedly during Omicron BA.2 dominance, whereas the protection against severe COVID-19 associated with at least three doses remained at high levels.

**Table 2:** Protection against severe COVID-19 associated with vaccination status with or without prior SARS-CoV-2 infection^a^ in each follow-up period during SARS-CoV-2 Omicron variant dominance, Scania county, Sweden, 2021 week 52 – 2022 week 11 (n = 593 cases; n = 5,930 controls)

Vaccination and infection history	Follow-up period						
	Omicron BA.1 2021 w52–2022 w1		Transition 2022 w2–3			Omicron BA.2 2022 w4–11	
	Protection	95% CI^b^	Protection		95% CI^b^	Protection	95% CI^b^
0 – 1 dose	Reference		Reference			Reference	
0 – 1 dose + prior infection	81	0–98	85	0–98		54	0–87
2 doses	91	79–96	70	41–85		57	17–78
2 doses + prior infection	91	57–98	92	59–98		53	0–82
≥ 3 doses	94	83–98	92	83–96		82	64–91
≥ 3 doses + prior infection	100	NA^c^	85	47–96		88	64–96

CI: confidence interval; COVID-19: coronavirus disease; OR: odds ratio; SARS: severe acute respiratory syndrome coronavirus 2.

^a^ SARS-CoV-2 infections included were at least 90 days before the case date recorded in the study period.

^b^ Protection was estimated as 100 x (1– OR).

^c^ Not possible to estimate CI.

Estimates were obtained from conditional logistic regression with adjustment for comorbidities (0, 1, ≥ 2).

The following vaccines were used during the study period: Comirnaty (BNT162b2 mRNA, BioNTech-Pfizer), Spikevax (mRNA-1273, Moderna), Vaxzevria (ChAdOx1-S, AstraZeneca).

## Discussion

The main finding of the present study was the marked decline in protection against severe COVID-19 during Omicron BA.2 dominance among persons with two vaccine doses only. The decline occurred quite rapidly over two months and thus cannot be explained by waning VE alone. A more likely explanation is increased immune evasiveness properties of BA.2, which gives this genetically distinct subvariant a competitive advantage in highly vaccinated populations [1]. The relatively stable protection among individuals with at least three doses during follow-up in our study suggests that a very robust immune response is necessary to confer protection also against BA.2.

Although the overall vaccination uptake of two doses among adults is high (83%) in the study cohort; only 57% had accepted the booster dose that was generally offered to everyone in Scania by the end of the study period. The Scania region is ethnically and socioeconomically diverse and we have previously reported substantially lower vaccination uptake among population groups born outside Sweden [7]. In the current study, the overrepresentation of foreign-born individuals among cases with severe disease decreased during the transition from BA.1 to BA.2, which suggests more widespread dropouts from the vaccination programme after two doses also among people born in Sweden. This means that the overall population protection against severe COVID-19 can be insufficient in case of new intermittent SARS-CoV-2 epidemics that may become the ‘new normal’ [8].

Another notable finding was that the protection associated with a prior SARS-CoV-2 infection also declined after the transition from Omicron BA.1 to BA.2. A recent total population study from Sweden reported long-lasting protection among unvaccinated persons who have survived and recovered from a previous SARS-CoV-2 infection [9]. However, the follow-up of that study ended before Omicron became dominant. Our study, with a more recent follow-up, suggests that the natural protection from previous VOCs against severe disease is markedly lower when Omicron BA.2 is dominant.

The key strengths of our study were the detailed individual-level data on vaccinations, infections and hospitalisations and the possibility to stratify hospitalised people further by disease severity, thereby limiting the misclassification of cases hospitalised with – rather than because of – COVID-19. A major limitation was that we lacked data on the virus variant for the individual cases in each time period. Our study may therefore underestimate the true change in VE associated with the transition from BA.1 to BA.2. Another limitation was that we could not evaluate protection from prior Omicron infection, as the follow-up period with Omicron dominance is still short. Evidence from Qatar suggests that infection from BA.1 protects against reinfection with BA.2 but this was observed during a very short follow-up period [10]. A further limitation of our study was that general testing was no longer generally recommended in Sweden, which means that VE against infection could not be evaluated. It should also be noted that the statistical uncertainty in some of our subgroups was substantial, as reflected by the wide and overlapping CIs across the follow-up periods. Continued monitoring of VE associated with Omicron BA.2 is therefore warranted.

## Conclusion

VE remained relatively stable after the transition from the Omicron BA.1 to BA.2 subvariant among people with at least three doses but point estimates decreased markedly among those with only two doses. Protection from prior infection was also lower when BA.2 was dominant. These findings from our population-based study suggest that booster vaccination is needed to maintain sufficient protection against severe COVID-19.

## Supplementary Data

246000 Supplement
