# Supplementary material for: COVID-19 vaccine effectiveness against severe disease from SARS-CoV-2 Omicron BA.1 and BA.2 subvariants – surveillance results from southern Sweden, December 2021 to March 2022
Source: Euro Surveill. 2022 May 5;27(18):2200322. doi: 10.2807/1560-7917.ES.2022.27.18.2200322 (PMC9074397; doi:10.2807/1560-7917.ES.2022.27.18.2200322)
Supplement: Supplement [file 22-00322_BJORK_Supplement.pdf]

Supplementary Material

COVID-19 vaccine effectiveness against severe disease from the Omicron BA.1 and BA.2 subvariants – surveillance results from southern Sweden, December 2021 to March 2022

Jonas Björk[1,2], Carl Bonander[3], Mahnaz Moghaddassi[4], Magnus Rasmussen[5], Ulf Malmqvist[2], Malin Inghammar[5], Fredrik Kahn[5]

[1]Clinical Studies Sweden, Forum South, Skåne University Hospital, Lund, Sweden
[2]Division of Occupational and Environmental Medicine, Lund University, Lund, Sweden
[3]School of Public Health and Community Medicine, Institute of Medicine, University of Gothenburg, Sweden
[4]Social Medicine and Global Health, Department of Clinical Sciences Malmö, Lund University, Malmö, Sweden
[5]Department of Clinical Sciences Lund, Section for Infection Medicine, Skåne University Hospital, Lund University, Lund, Sweden

Content

Supplementary Table S1
Supplementary Table S2
Supplementary Table S3

This supplementary material is hosted by Eurosurveillance as supporting information alongside the article *COVID-19 vaccine effectiveness against severe disease from the Omicron BA.1 and BA.2 subvariants – surveillance results from southern Sweden, December 2021 to March 2022*, on behalf of the authors, who remain responsible for the accuracy and appropriateness of the content. The same standards for ethics, copyright, attributions and permissions as for the article apply. Supplements are not edited by Eurosurveillance and the journal is not responsible for the maintenance of any links or email addresses provided therein.

Supplementary Table S1. Routine sequencing of samples of infected cases, Scania, Sweden, 2021 w52 – 2022 w11 (n = 2,999).

Year	2021	2022										
Week	52	1	2	3	4	5	6	7	8	9	10	11
Total	289	325	281	340	288	314	314	222	222	123	124	157
Omicron BA.1	162	206	136	153	72	65	40	32	77	6	6	6
Omicron BA.2	29	61	124	179	214	244	273	190	144	117	118	151
Delta	98	58	21	8	2	5	1	0	1	0	0	0

Supplementary Table S2. Classification of comorbidities.

Disease group	ICD-10 codes (incl KVÅ-codes[a])
Cardiovascular diseases	I10-I15, I20-I25, I42-I43<br>I50, I60-I69<br>J81
Diabetes or obesity	E10, E11, E66
Kidney or liver diseases	K70.X, K74.3-K74.6, K75.4, K76.0<br>N18.5, N18.9<br>DR016, DR024
Respiratory diseases	A15-A19<br>E84<br>I26, I27<br>J42, J43, J44, J45, J47, J84<br>J96, J98.2, J98.3
Neurological diseases (including dementia)	G00-G99<br>F00-F03
Cancer or immunosuppressed state (including organ transplantation)	C00-C99<br>KAS, FQA, FQB, JJC, GDG, JLE<br>DR046, DR047, DR048<br>D80.0-D80.1<br>D80.5, D81, D82, D83
Other conditions and diseases<br>• HIV<br>• Thalassemia<br>• Sickle cell<br>• Mood disorders<br>• Schizophrenia spectrum disorders<br>• Substance use disorders<br>• Downs syndrome	B20-B24<br>D56, D57<br>F10-F19, F30-F39, F20-F29<br>Q90

[a] Swedish classification of certain interventions during health care visits

Supplementary Table S3. Vaccine effectiveness after at least three or two doses against severe COVID-19 in each follow-up period, Scania, Sweden, 2021 w52 – 2022 w11 (n = 593 cases; n = 5,930 controls).

	Follow up period		
	Omicron BA.1<br>2021 w52-2022 w1	Transition<br>2022 w2-3	Omicron BA.2<br>2022 w4-11
	VE (95% CI)[a]	VE (95% CI)[a]	VE (95% CI)[a]
At least three doses			
All	94 (84 – 98)	90 (82 – 95)	82 (64 – 91)
Age			
< 65 years	92 (73 – 98)	74 (16 – 92)	76 (32 – 91)
≥ 65 years	94 (76 – 98)	92 (83 – 97)	82 (56 – 93)
Sex			
Females	92 (74 – 97)	93 (81 – 98)	78 (35 – 93)
Males	96 (79 – 100)	89 (74 – 96)	84 (62 – 93)
Comorbidities			
None	99 (53 – 100)	96 (81 – 100)	77 (27 – 92)
≥ 1	90 (44 – 98)	84 (54 – 95)	85 (48 – 96)
Two doses			
All	90 (78 – 95)	70 (42 – 84)	54 (13 – 75)
Age			
< 65 years	93 (81 – 98)	78 (35 – 93)	59 (4 – 83)
≥ 65 years	84 (37 – 96)	55 (0 – 81)	43 (0 – 79)
Sex			
Females	88 (65 – 96)	73 (37 – 88)	53 (0 – 82)
Males	92 (73 – 98)	67 (14 – 87)	54 (0 – 80)
Comorbidities			
None	96 (66 – 100)	79 (32 – 94)	61 (2 – 85)
≥ 1	77 (0 – 95)	58 (0 – 86)	46 (0 – 86)

[a]Vaccine effectiveness (95% confidence interval). Estimates were obtained from conditional logistic regression for age and sex matched case and controls (1:10), and with adjustment for comorbidities (0, 1, ≥2) and infection at least 90 days prior the case date. Results are presented overall and stratified by age, sex and comorbidities.
